# Design of testbed and preliminary data for de-icing experiments using piezoelectric actuators

**DOI:** 10.1016/j.dib.2018.07.050

**Published:** 2018-07-26

**Authors:** Dongkyoung Lee, Dahoon Ahn

**Affiliations:** a Department of Mechanical and Automotive Engineering, Kongju National University, Cheonan 31080, Republic of Korea; b Advanced Railroad Vehicle Division, Korea Railroad Research Institute, Uiwang-si, Gyeonggi-do, Republic of Korea

## Abstract

The piezoelectric actuators, providing mechanical energy, are a good option to remove the ice efficiently. Therefore, a testbed for experiments are designed and the preliminary data of de-icing phenomena is provided. An ice specimen, formed from sterilized distilled water under −30 °C, is detached from the base materials after the voltage signal is engaged. The parameterized detachment phenomena and the analysis data are presented. The independent variables are ice thickness and frequency of the voltage input. The detachment distance is presented depending on the independent variables.

**Specifications Table**

**Table:** 

Subject area	*Mechanical engineering, Applied physics,*
More specific subject area	*Piezoelectric actuator, de-icing, ice fracture*
Type of data	*Numerical value, figure*
How data was acquired	*Piezoelectric actuator (PICMA® PL088.31, Physik Instrument, Germany), Base material (SMA490A, Steelmax, Korea), Piezo controller(MDT694B, Thorlabs, NJ, USA), Function generator (33521A, Keysight Technologies, CA, USA), Sterilized distilled water (Sterilized distilled water for perfusion, JW Pharmaceutical, Korea), freezer (LOC-251F, Lassele, Korea)*
Experimental factors	*Voltage, Amplitude, and Period of Square wave; Temperature of icing.*
Experimental features	*Ice is formed from Sterilized distilled water under −30 °C. Electric voltage signal is engaged and ice is detached from the base material.*
Data format	*Raw and analyzed*
Data source location	*Cheonan, South Korea*
Data accessibility	*Dataset is within this article*

**Value of the data**

• Experimental testbed design can be easily accessed for mechanical de-icing research in the various fields.
• Information of experimental equipment can be recognized and experimental testbed can be easily established.
• Dataset presented in this article provides a reference to de-icing experiments using piezoelectric actuators.
• Detachment phenomena can be compared to other de-icing experiments or experimental conditions.

## 1 Data

**Fig. 1:**
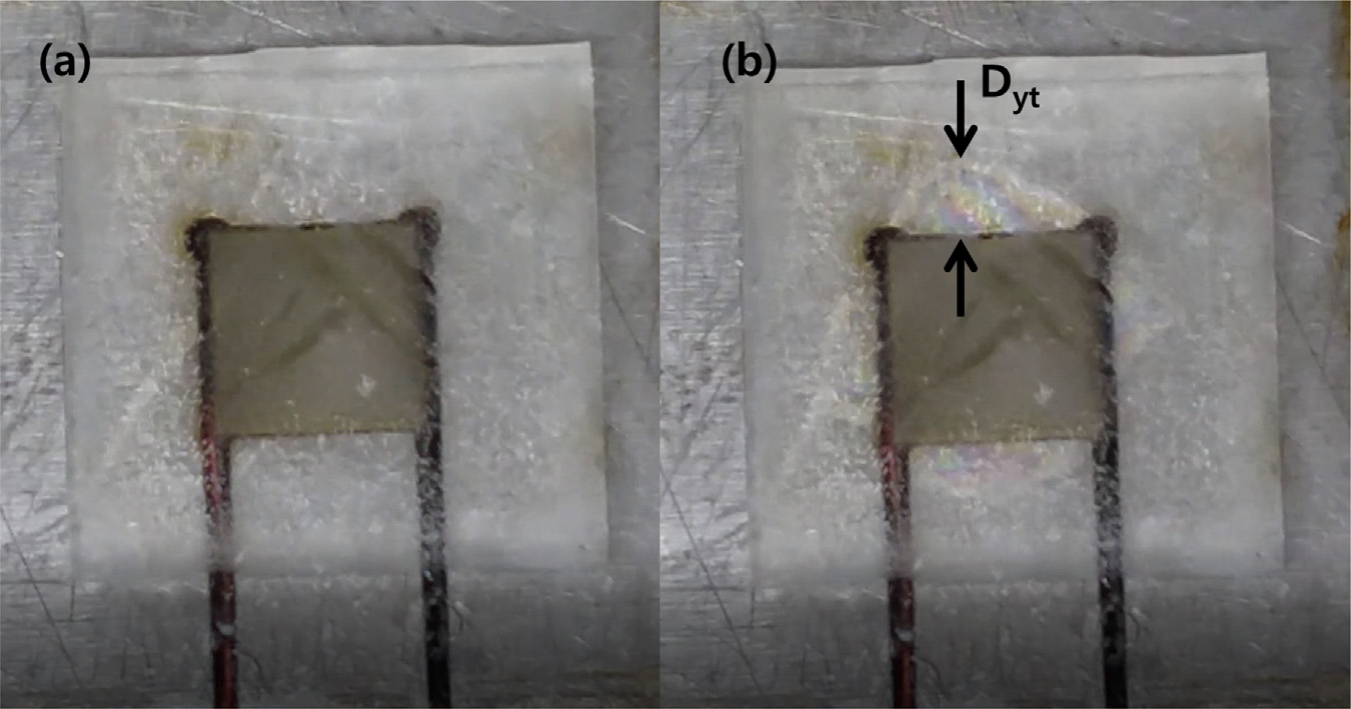
Top view of the detachment. (a) Before engaging voltage signal (b) right after engaging voltage signal with the detachment distance.

**Fig. 2:**
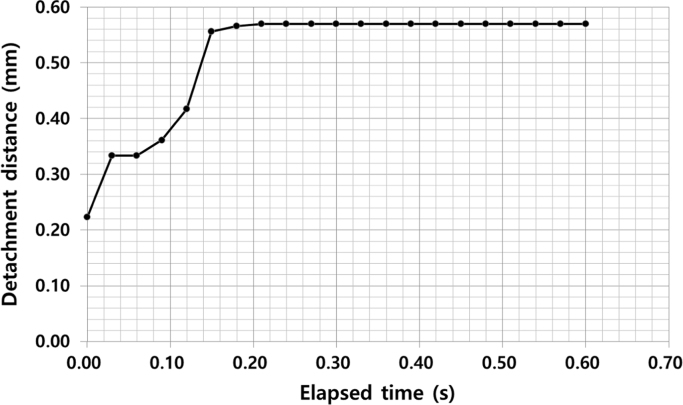
Detachment distances depending on time.

**Fig. 3:**
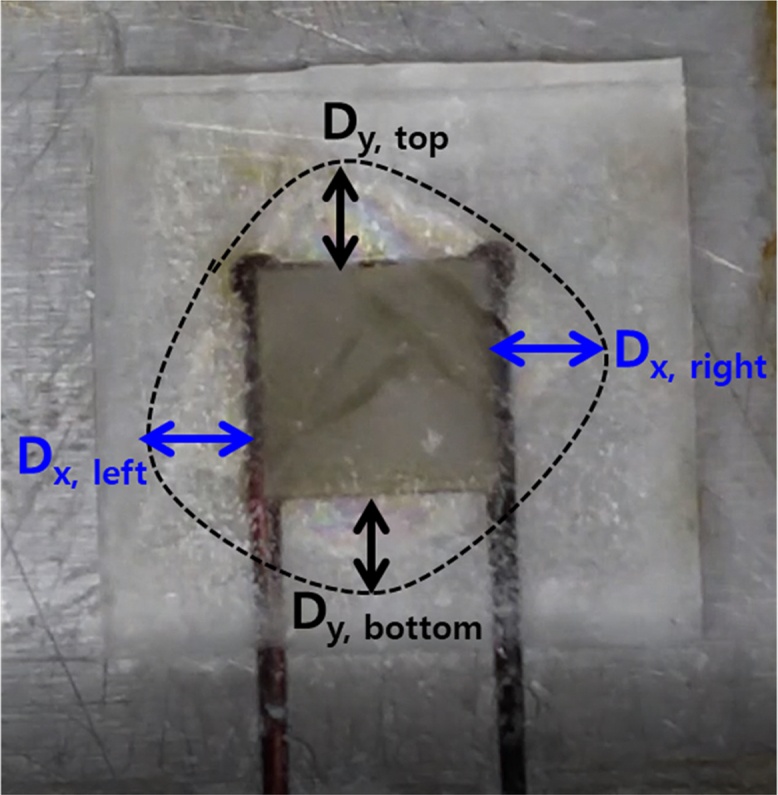
Depiction of detachment distance defined from four areas.

**Fig. 4:**
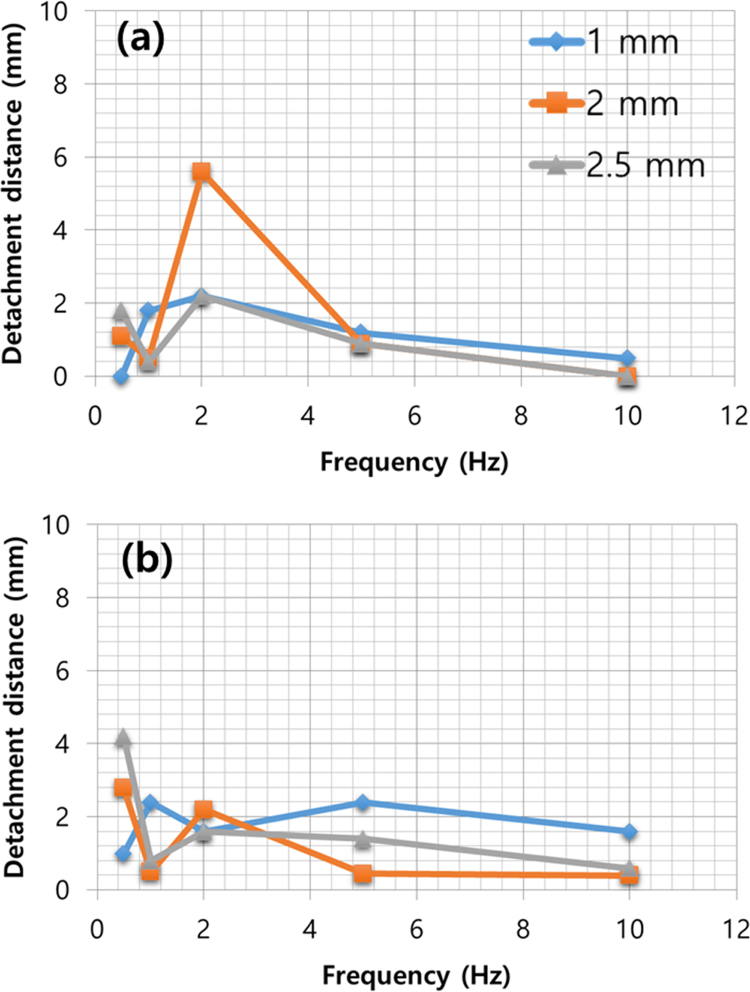
Detachment distance in (a) $D_{y,\;\mathrm{top}}$ and (b) $D_{y,\;\mathrm{bottom}}$.

**Fig. 5:**
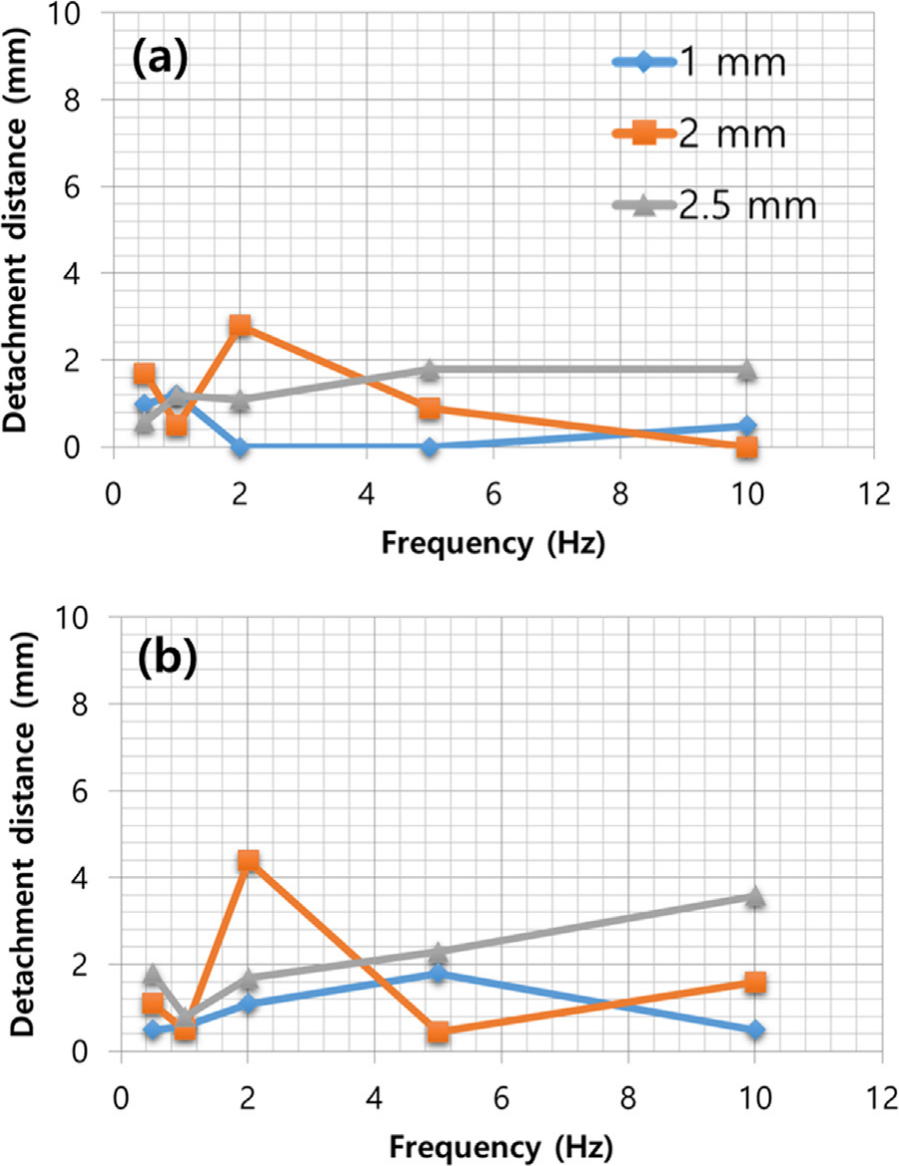
Detachment distance in (a) $D_{x,\;\mathrm{left}}$ and (b) $D_{x,\;\mathrm{right}}$.

The experimental parameters are tabulated in Table 1. The input voltage signal is square wave, and both of the amplitude and the offset are 50 V. Thus, the minimum and maximum value of the signal are 0 V and 100 V, respectively. The dimension and travel range of the piezoelectric actuator is 10 mm(Width)×10 mm (Length)×2 mm (Height) and 2.2 μm, respectively. The ice specimen formed from sterilized distilled water and its dimension is 25 mm (Width)×25 mm (Length). Top view of the ice specimen before and after the voltage signal engagement is shown in Fig. 1(a) and (b), respectively. The detachment distance in terms of elapsed time is shown in Fig. 2, when the thickness of the ice specimen is 2 mm and the frequency of the voltage input is 0.5 Hz. The detachment distance is 0.22 mm right after the voltage signal is engaged (*t*=0 s). This distance increases from 0.22 to 0.57 mm in 0.18 s and then the distance is saturated. A dataset of detachment distance depending on the independent variables such as ice thickness and frequency of the voltage input is presented. The detachment distance can be defined from four areas and its definition is shown in Fig. 3. The dataset of measured detachment distance is tabulated in Table 2. The dataset shown in Table 2 is plotted in terms of frequency and shown in Figs. 4 and 5.

**Table 1:** Experimental parameters.

**Parameter**	**Value**
Piezoelectric actuator dimension (mm)	10 (W)×10 (L)×2 (H)
Piezoelectric actuator travel range (μm)	2.2
Ice specimen dimension (mm)	25 (W)×25 (L)
Voltage signal shape	Square wave
Maximum voltage (V)	100
Minimum voltage (V)	0

**Table 2:** Experimental design using independent variables and detachment distance as results.

**Variables**		**Detachment distance**			
		***x*-direction (mm)**		***y*-direction (mm)**	
**Thickness (mm)**	**Frequency (Hz)**	$D_{x,\mathrm{left}}$	$D_{x,\mathrm{right}}$	$D_{y,\mathrm{bottom}}$	$D_{y,\mathrm{top}}$
1	0.5	1	0.5	1	1,1
	1	1.2	0.6	2.4	1.8
	2	0	1.1	1.6	2.2
	5	0	1.8	2.4	1.2
	10	0.5	0.5	1.6	0.5
2	0.5	1.7	1.1	2.8	1.1
	1	0.5	0.5	0.5	0.5
	2	2.8	4.4	2.2	5.6
	5	0.9	0.45	0.45	0.9
	10	0	1.6	0.4	0
2.5	0.5	0.6	1.8	4.2	1.8
	1	1.2	0.8	0.8	0.4
	2	1.1	1.7	1.6	2.2
	5	1.8	2.3	1.4	0.9
	10	1.8	3.6	0.6	0

## 2 Experimental design, materials and methods

### 2.1 Testbed design of de-icing experiments

**Fig. 6:**
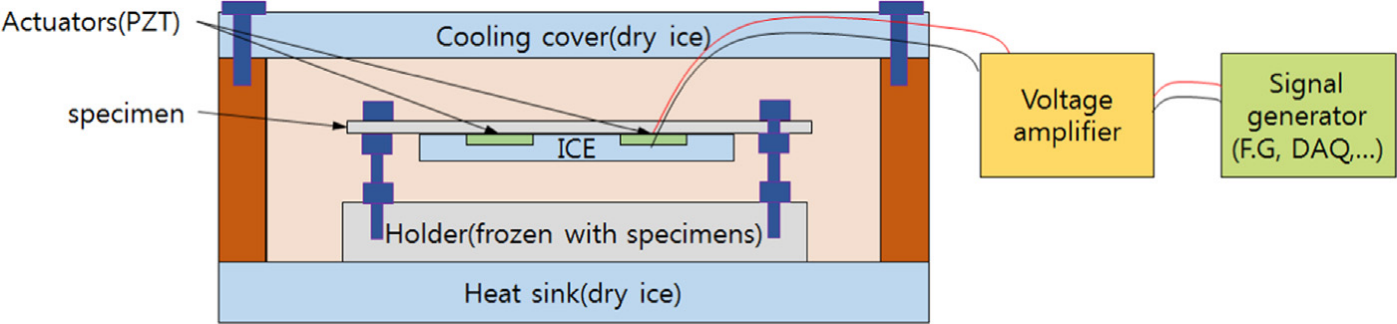
The schematic of de-icing experiments using piezoelectric actuators.

The schematic of the experimental set-up is shown in Fig. 6. An ice specimen with a piezoelectric actuator is placed into a refrigerator. The refrigerator is surrounded by the cooling cover and the heat sink for insulation. The externally connected function generator provides a voltage signal to the piezoelectric actuator via a voltage amplifier. Various signals can be generated from the function generator by controlling the variables. This signal can be used as independent variables.

### 2.2 Experiment procedure

**Fig. 7:**
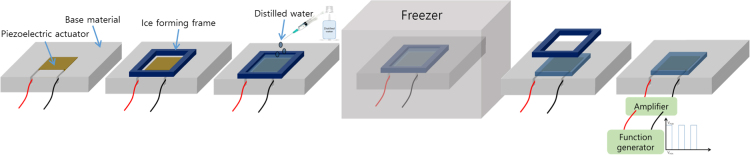
Experimental procedure.

The experiment procedure is shown in Fig. 7. The piezoelectric actuator is placed on the base material. In order to eliminate the height difference, the base material is machined in depth by the thickness of the piezoelectric actuator. An ice forming frame is placed on the base material and the sterilized distilled water is poured. Then, this is placed in the freezer under the temperature of −30 °C*.* After 3 h later, the ice is formed and the ice forming frame is detached. An electric voltage signal is provided and then the detachment phenomena are observed.

### 2.3 Measurement

Once the input voltage signal is engaged, the ice specimen is detached from the base material. Detached shape looks like rhombus shape. The detachment distance is defined from four areas. First, the detachment distance from the top edge of the piezoelectric actuator in the perpendicular and outward direction is defined by $D_{y,\mathrm{top}}$. Second, the detachment distance from the bottom edge of the piezoelectric actuator in the perpendicular and outward direction is defined by $D_{y,\mathrm{bottom}}$. The detachment distance from the left and right edge of the piezoelectric actuator in the perpendicular and outward direction are defined by $D_{x,\mathrm{left}}$ and $D_{x,\mathrm{right}}$, respectively. Based on these definitions, the detachment distance is measured right after the voltage input is engaged. The experimental parameters set as constant are shown in Table 1. Independent variables used in the experiments are tabulated in Table 2. Thickness of the ice specimen is changed in 1, 2, and 2.5 mm. The frequency of voltage signal is varied in 0.5, 1, 2, 5, and 10 Hz. Top view with the detachment distance in x- and y-direction is shown in Fig. 7.

