# Supplementary material for: Design of testbed and preliminary data for de-icing experiments using piezoelectric actuators
Source: Data Brief. 2018 Jul 26;20:146–51. doi: 10.1016/j.dib.2018.07.050 (PMC6091316; doi:10.1016/j.dib.2018.07.050)
Supplement: Supplementary file 1 — Supplementary material [file mmc1.docx]

**Title:** **Design of testbed and preliminary data for de-icing experiments using piezoelectric actuators**

The authors whose names are listed immediately below certify that they have NO affiliations with or involvement in any organization or entity with any financial interest (such as honoraria; educational grants; participation in speakers’ bureaus; membership, employment, consultancies, stock ownership, or other equity interest; and expert testimony or patent-licensing arrangements), or non-financial interest (such as personal or professional relationships, affiliations, knowledge or beliefs) in the subject matter or materials discussed in this manuscript.

**Author names:**

**Dongkyoung Lee and Dahoon Ahn**
